# The prognostic and predictive role of tumor-infiltrating lymphocytes (FoxP3 + and CD8 +) and tumor-associated macrophages in early HER2 + breast cancer

**DOI:** 10.1007/s10549-023-07017-8

**Published:** 2023-07-10

**Authors:** Minna M. Jääskeläinen, Satu Tiainen, Hanna Siiskonen, Maarit Ahtiainen, Teijo Kuopio, Aino Rönkä, Tiia Kettunen, Kirsi Hämäläinen, Kirsi Rilla, Ilkka Harvima, Arto Mannermaa, Päivi Auvinen

**Affiliations:** 1 Cancer Center, Kuopio University Hospital, Northern Savonia Healthcare Municipality, P.O.Box 100, 70029 Kuopio, Finland; 2 Institute of Clinical Medicine, University of Eastern Finland, Kuopio, Finland; 3 Imaging Center, Clinical Pathology, Kuopio University Hospital, Northern Savonia Healthcare Municipality, Kuopio, Finland; 4 Department of Pathology, Central Finland Hospital Nova, Jyväskylä, Finland; 5 Department of Biological and Environmental Science, University of Jyväskylä, Jyväskylä, Finland; 6 Institute of Clinical Medicine, Clinical Pathology and Forensic Medicine, University of Eastern Finland, Kuopio, Finland; 7 Biocenter Kuopio and Cancer Center of Eastern Finland, University of Eastern Finland, Kuopio, Finland; 8 Institute of Biomedicine, University of Eastern Finland, Kuopio, Finland; 9 Department of Dermatology, Kuopio University Hospital, Northern Savonia Healthcare Municipality and University of Eastern Finland, Kuopio, Finland; 10 Biobank of Eastern Finland, Kuopio University Hospital, Northern Savonia Healthcare Municipality, Kuopio, Finland

**Keywords:** Tumor-infiltrating lymphocytes, Tumor-associated macrophages, FoxP3, CD8, Breast cancer, HER2

## Abstract

**Purpose:**

In HER2-positive (HER2 +) breast cancer, tumor-infiltrating lymphocytes (TILs) and tumor-associated macrophages (TAMs) may influence the efficacy of the HER2-antibody trastuzumab and the patient’s outcome. In this HER2 + patient cohort, our aim was to study the numbers of FoxP3 + regulatory TILs and CD8 + cytotoxic TILs, their correlations with CD68 + and CD163 + TAMs, and the prognostic and predictive value of the studied factors.

**Methods:**

We evaluated 139 non-metastatic HER2 + breast cancer patients operated between 2001 and 2008. The FoxP3+TIL count (FoxP3+TILs) was assessed using the hotspot method, and the CD8 + TIL count (CD8+mTILs) utilizing a digital image analysis from invasive margin areas. The ratios between CD8+mTILs and FoxP3+TILs as well as CD8+mTILs and TAMs were calculated.

**Results:**

FoxP3 + TILs and CD8 + mTILs correlated positively with each other (p<0.001). FoxP3+TILs had a positive correlation with CD68+and CD163+TAMs (p≤0.038), while CD8 + mTILs correlated only with CD68+TAMs (p<0.001). In the HER2 + and hormone receptor-positive Luminal B subgroup, high numbers of FoxP3+TILs were associated with shorter disease-free survival (DFS) (54% vs. 79%, p = 0.040). The benefit from adjuvant trastuzumab was extremely significant among patients with a high CD8 + mTILs/CD68 + TAMs ratio, with overall survival (OS) 84% vs. 33% (p = 0.003) and breast cancer-specific survival (BCSS) 88% vs. 48% (p = 0.009) among patients treated with or without trastuzumab, respectively.

**Conclusion:**

In the HER2 + Luminal B subgroup, high FoxP3 + TILs were associated with shorter DFS. A high CD8 + mTILs/CD68 + TAMs ratio seems to associate with impressive efficacy of trastuzumab.

**Supplementary Information:**

The online version contains supplementary material available at 10.1007/s10549-023-07017-8.

## Introduction

The human epidermal growth factor receptor 2 (HER2) gene is amplified in 15–20% of breast cancers leading to uncontrolled cell proliferation as a result of overexpression of the HER2-receptor in breast cancer cells [1]. HER2-positive (HER2 +) breast cancers are biologically aggressive but anti-HER2 treatments, such as trastuzumab, have significantly improved their prognosis [1, 2]. Unfortunately, some patients still do not gain long-lasting benefits from the current treatments. Therefore, it is crucial to clarify the mechanisms behind the tumor progression and resistance to these drugs.

Trastuzumab restricts the progression of HER2 + breast cancer in several ways. The binding of trastuzumab to the extracellular domain of HER2-receptor subsequently blocks the actions of its tyrosine kinase component which suppresses the proliferation of malignant cells [1]. Furthermore, trastuzumab can trigger an immune response against cancer cells via antibody-dependent cytotoxicity, i.e., an immune response is activated when trastuzumab binds to its receptor on the cell’s surface [1, 3]. Thus, it can be hypothesized that the presence of immune cells in the tumor microenvironment may contribute to the clinical benefit derived from trastuzumab and consequently to the patient’s outcome. While in some studies, high numbers of tumor-infiltrating lymphocytes (TILs) have been associated with an increased efficacy of trastuzumab in early HER2 + breast cancer [4] also contrasting results have been reported [5], and thus it is important to understand the interplay between different immune cells in the tumor microenvironment.

It is interesting that high levels of TILs are found especially in the aggressive breast cancer subtypes, such as triple-negative and HER2 + breast cancer [6]. Among these patient subgroups, a high level of TILs correlates with a favorable clinical outcome [7–9]. While cytotoxic CD8 + T-cells possess various properties that inhibit cancer growth, there are other T-cells, such as regulatory T-cells (Tregs) which promote immunosuppressive conditions that may even enhance cancer progression [10]. Furthermore, tumor-associated macrophages (TAMs), particularly cells with the M2-like phenotype, participate in creating pro-tumoral inflammatory conditions. There are reports that a high level of TAMs is associated with a poor outcome in breast cancer [11–13].

Previous studies have provided information of the prognostic value of TILs in breast cancer but the role of different TIL subtypes and their interactions with other immune cell types such as TAMs are less well defined. The aim of this study was to investigate the prognostic and predictive role of FoxP3 + regulatory TILs, CD8 + cytotoxic TILs in the tumor margin (CD8 + mTILs), and the ratios of CD8 + mTILs with FoxP3 + TILs and TAMs in a material of 139 non-metastatic HER2 + breast cancer patients. Approximately half of the patients had been treated before trastuzumab became available as an adjuvant treatment of HER2 + breast cancer, while the remainder of the patients received adjuvant trastuzumab. Since the predictive value of TILs in trastuzumab therapy has remained controversial, the special aim of this study was to investigate if the studied immune cells or their ratios would have any role in predicting the efficacy of adjuvant trastuzumab therapy.

## Material and methods

### Patient material

The study population consisted of 139 primary, non-metastatic HER2 + (chromogenic in situ hybridization (CISH) positive) breast cancer patients operated in the Kuopio University Hospital between 2001 and 2008 and for whom adequate tumor material was available. The collection of the primary patient material has been described previously [14]. Adjuvant treatments including chemotherapy and trastuzumab were administered according to Finnish national guidelines. Survival follow-up was updated on May 26th 2020. The study was conducted according to the Declaration of Helsinki, and the University of Eastern Finland Ethics Committee issued the ethical approval (February 24, 2009, 19//2009).

### Assessment of FoxP3 + TILs

FoxP3 immunoreactivity was considered as an indicator for Tregs [15, 16]. Formalin-fixed, paraffin-embedded sections of primary breast cancer specimens were immunohistochemically stained for FoxP3 + TILs with an anti-FoxP3 antibody (1:300, monoclonal mouse antibody clone 236A/E7, Abcam, UK). The marker proteins were visualized with the ABC technique using diaminobenzidine, and nuclei were counterstained with hematoxylin. In each staining group, a section of tonsil was included as a positive control, while a breast cancer sample omitting the primary antibody served as a negative control.

Those areas with the highest density of FoxP3 + TILs (hot spots) were first detected visually by scanning the section at low magnification, followed by examination of the hot spots in the invasive area and in the stroma near the invasive area in greater detail. The number of positive cells in the hot spots was counted in at least three high-power fields (× 40 objective) and averaged to represent the FoxP3 + TIL count in the section.

### Assessment of CD8 + TILs

Formalin-fixed, paraffin-embedded sections of tumor material from patients undergoing breast cancer surgery were immunohistochemically stained for CD8 + TILs with an anti-CD8 antibody (1:400, rabbit monoclonal antibody, RM-9116-S (SP16), Thermo Scientific, USA). The marker proteins were visualized with diaminobenzidine, and nuclei counterstained with hematoxylin. A section of tonsil was used as a positive control.

The stained whole-section slides were digitally scanned using a NanoZoomer-XR (Hamamatsu Photonics) with a × 40 objective. CD8 + TIL counts were calculated from manually selected representative areas of the invasive tumor margin with the digital image analysis software QuPath [17]. According to the recommendation of the International Immuno-oncology Biomarkers Working Group [18], the invasive margin was defined as an area centered on the tumor border with a width of 1 mm. Depending on the size of the adequately stained invasive cancer, the mean analyzed area from the invasive margin was 5.3 mm^2^ (range 2.3–8.5 mm^2^).

### Assessment of TAMs and the standard histopathological parameters

Immunohistochemical stainings and the assessment of TAMs were performed as described previously [12]. CD68- and CD163-positivity indicated all TAMs and M2-like TAMs, respectively.

The TNM classification was assessed by a pathologist according to the international guidelines [19]. The threshold for estrogen receptor (ER) and progesterone receptor (PR) positivity was > 10%, and cases were defined as hormone receptor-positive (HR +) when either ER or PR was positive. The HER2 gene amplification was analyzed by the CISH test; the threshold for HER2 positivity was six or more gene copies per nucleus.

### Statistical methods

IBM SPSS Statistics version 26 (IBM Corporation, Armonk, NY, USA) was utilized in the statistical analyses. FoxP3 + TIL and CD8 + mTIL counts, and the ratios of CD8 + mTILs with the other investigated immune cells i.e., CD8 + mTILs/FoxP3 + TILs, CD8 + mTILs/CD68 + TAMs and CD8 + mTILs/CD163 + TAMs were graded as low or high according to the median. Chi-square test, Kruskall-Wallis test, and Mann–Whitney U-test were used for calculating the differences between the investigated factors, Spearman rank correlation for the correlations between the investigated immune cells, Cox’s model for survival analyses and the Kaplan Meier method for visual representation of the survival curves. For overall survival (OS) death from any cause, for breast cancer-specific survival (BCSS) death from breast cancer, and for disease-free survival (DFS) any breast cancer recurrence, were counted as an event. P-values < 0.05 were regarded as statistically significant.

## Results

**Fig. 1:**
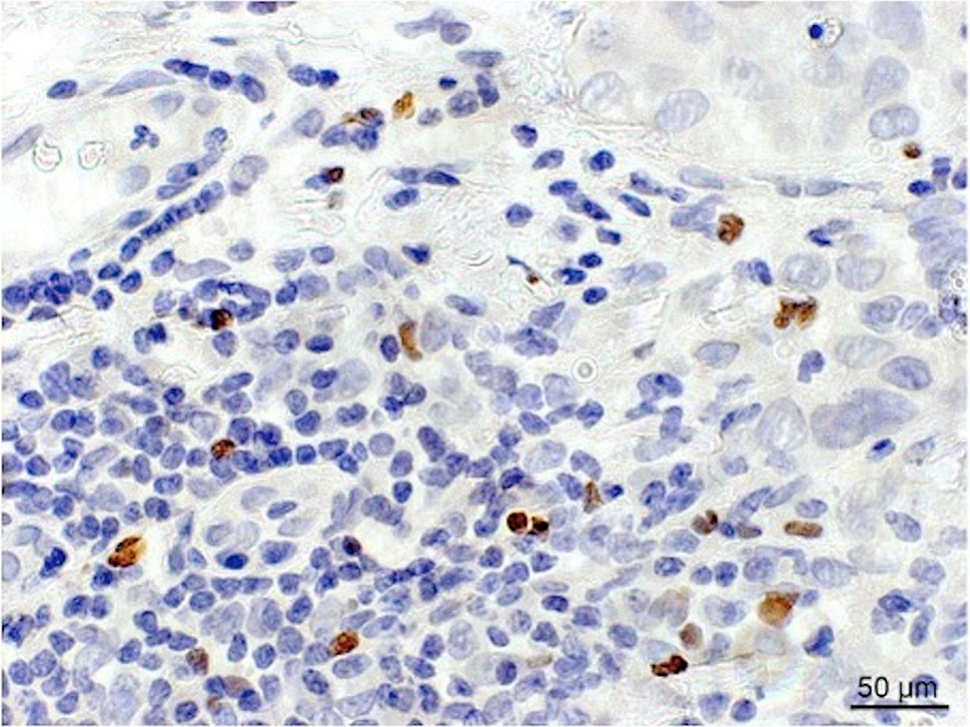
Example of FoxP3 + TILs immunostaining. The figure shows an example of a breast cancer section stained with the FoxP3 antibody

**Fig. 2:**
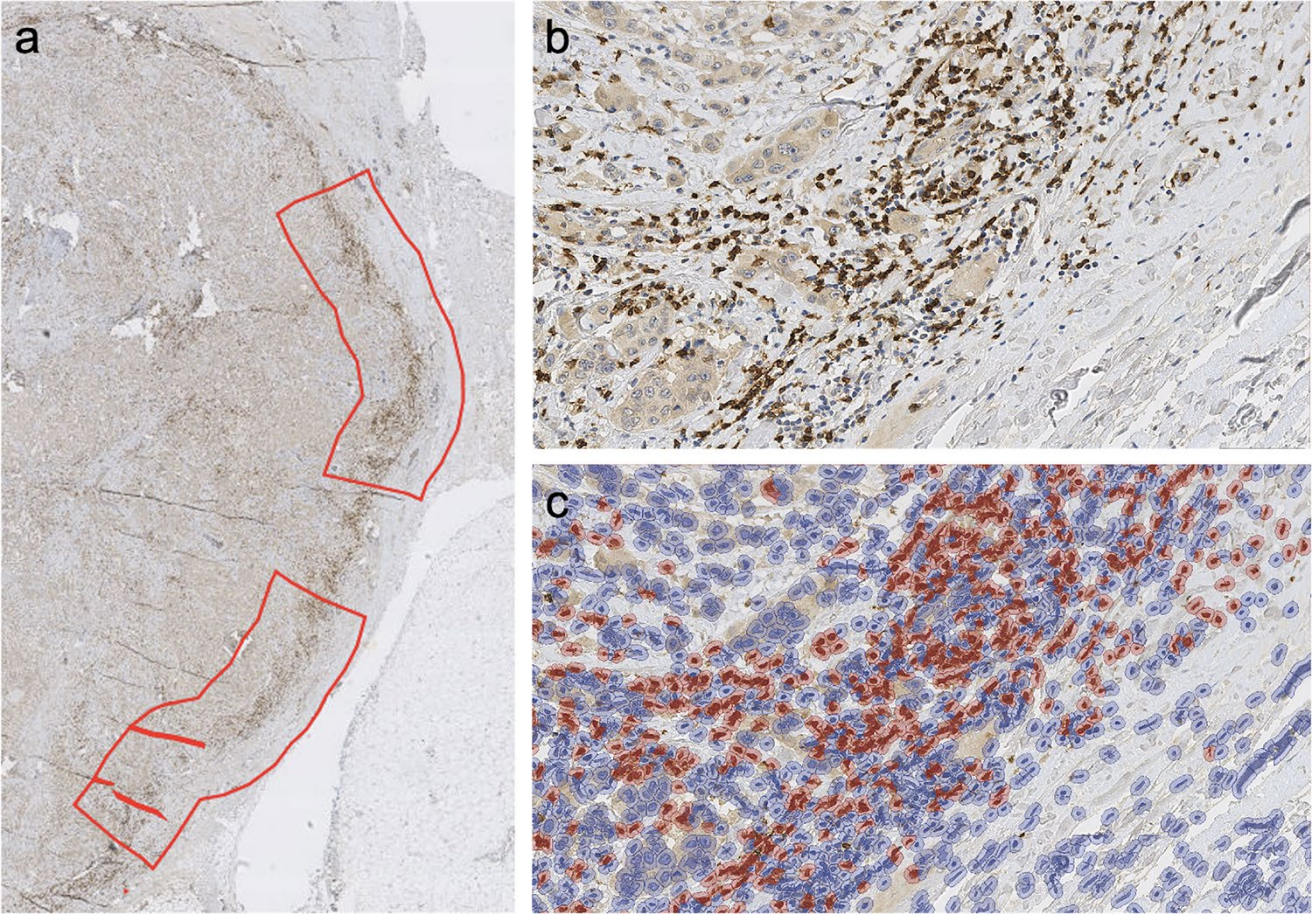
Example of calculating CD8 + TILs with QuPath. Representative areas of invasive tumor margin were manually selected to analyze the CD8 + TIL counts (panel **a**). A closer view of CD8 + TIL staining is shown in panel **b**. In panel **c**, the same area analyzed with digital image analysis software QuPath is shown, CD8 + TILs are marked with red

The FoxP3 + TIL count could be evaluated in 133 (96%) and the CD8 + TIL count in 106 (76%) cases. The median for the FoxP3 + TIL count assessed manually with the hot spot method was 25.7 (range 0–100*).* The median for the CD8 + TIL count in the tumor margin (CD8 + mTILs) as assessed with digital image analysis was 613.3/mm^2^ (range 14–2571). Examples of FoxP3 + and CD8 + TIL stainings and digital image analysis are shown in Figs. 1 and 2. The numbers of TAMs were determined previously [12], and in this HER2 + patient group, the median for CD163 + TAMs was 28.0 (range 8–64) and 38.5 (range 13–73) for CD68 + TAMs.

### Clinicopathological characteristics of the patients

The clinicopathological parameters of the 139 patients are presented in Table 1. Adjuvant trastuzumab was given to 63 (45%) patients, since the indication for provision of adjuvant anti-HER2-treatment was only included in national guidelines in the summer of 2005. In the updated clinical data, the median follow-up time was 12.7 years (range 0.4–18.3 years). During the follow-up, 53 patients (38%) experienced some form of relapse**,** 55 patients (40%) died**;** 43 of them due to breast cancer.

**Table 1:** Clinicopathological characteristics of the HER2-positive breast cancer patients

	All patients n = 139	Adjuvant trastuzumab n = 63	No adjuvant trastuzumab n = 76	p
Age, median (range)	58.7 (32–86)	53.1 (32.3–74.4)	66.0 (41.0–86.0)	< 0.001*
Adjuvant treatment, n (%)				
Chemotherapy	110 (79)	63 (100)	47 (62)	< 0.001*
Radiation therapy	124 (89)	59 (94)	65 (86)	0.124
Hormonal therapy	73 (53)	33 (52)	40 (53)	0.977
Tumor classification, n (%)				0.721
pT1	67 (48)	31 (49)	36 (47)	
pT2	60 (43)	25 (40)	35 (46)	
pT3	4 (3)	2 (3)	2 (3)	
pT4	8 (6)	5 (8)	3 (4)	
Nodal classification, n (%)				0.852
pN0	47 (34)	20 (32)	27 (36)	
pN1	55 (39)	26 (41)	29 (38)	
pN2	22 (16)	9 (14)	13 (17)	
pN3	15 (11)	8 (13)	7 (9)	
Histological grade, n (%)				0.483
1	4 (3)	1 (2)	3 (4)	
2	47 (34)	24 (38)	23 (30)	
3	88 (63)	38 (60)	50 (66)	
Histological type, n (%)				0.308
Ductal	119 (86)	55 (87)	64 (84)	
Lobular	9 (6)	2 (3)	7 (9)	
Other	11 (8)	6 (10)	5 (7)	
ER positive, n (%)	76 (55)	34 (54)	42 (55)	0.879
PR positive, n (%)	65 (47)	27 (43)	38 (50)	0.401
Any relapse, n (%)	53 (38)	16 (25)	37 (49)	0.005*
Distant metastases n (%)	45 (32)	12 (19)	33 (43)	0.002*
Death, n (%)	55 (40)	13 (21)	42 (55)	< 0.001*

*ER* estrogen receptor; *PR* progesterone receptor; *p < 0.05

High TIL counts, both FoxP3 + and CD8 + mTILs, were associated with a higher histological tumor grade (p ≤ 0.007). A high FoxP3 + TIL count was associated also with nodal involvement (p = 0.043), while a high CD8 + mTIL count was linked with PR negativity (p = 0.039). No statistically significant associations were found between FoxP3 + or CD8 + mTIL counts and other standard histopathological factors (data not shown).

### Correlations between TILs and TAMs in the tumor microenvironment

The FoxP3 + TIL count correlated positively with the CD8 + mTIL count (p < 0.001). When analyzing the correlations with TAMs, the FoxP3 + TIL count correlated positively with the numbers of CD68 + and CD163 + TAMs (p ≤ 0.038). The CD8 + mTIL count displayed a positive correlation with the numbers of CD68 + TAMs (p < 0.001), but not with CD163 + TAMs (Table 2).

**Table 2:** Spearman correlation coefficients (r) and p values for the relations of tumor-infiltrating lymphocytes (TILs) and tumor-associated macrophages (TAMs) in HER2-positive breast cancer samples

	FoxP3 + TILs		CD8 + mTILs	
	r	p	r	p
CD68 + TAMs	0.223	0.010*	0.416	< 0.001*
CD163 + TAMs	0.180	0.038*	0.052	0.599
CD8 + mTILs	0.442	< 0.001*		

*TILs* tumor-infiltrating lymphocytes; *TAMs* tumor-associated macrophages;

*CD8* + *mTILs* CD8 + TILs in the tumor margin; *p < 0.05

### Prognostic value of TILs

In the whole patient group, neither FoxP3 + TILs, CD8 + mTILs nor the ratios of CD8 + mTILs with FoxP3 + TILs or TAMs correlated with the outcome of the patients (data not shown). However, in HER2 + Luminal B (HER2 + and HR +) subgroup, a high FoxP3 + TIL count (n = 35) was associated with a shorter DFS (54% vs. 79%, HR 2.35, 95% CI 1.04–5.32, p = 0.040) and displayed also a non-significant trend towards inferior OS (57% vs. 74%, HR 1.73, 95% CI 0.79–3.77, p = 0.168) and BCSS (63% vs. 81%, HR 2.04, 95% CI 0.84–4.92, p = 0.113). When the analysis was restricted to the HER2 + /HR- cases, the FoxP3 + TIL count did not associate with the outcome of the patients (data not shown).

### Predictive value of CD8 + mTILs/CD68 + TAMs ratio

**Fig. 3:**
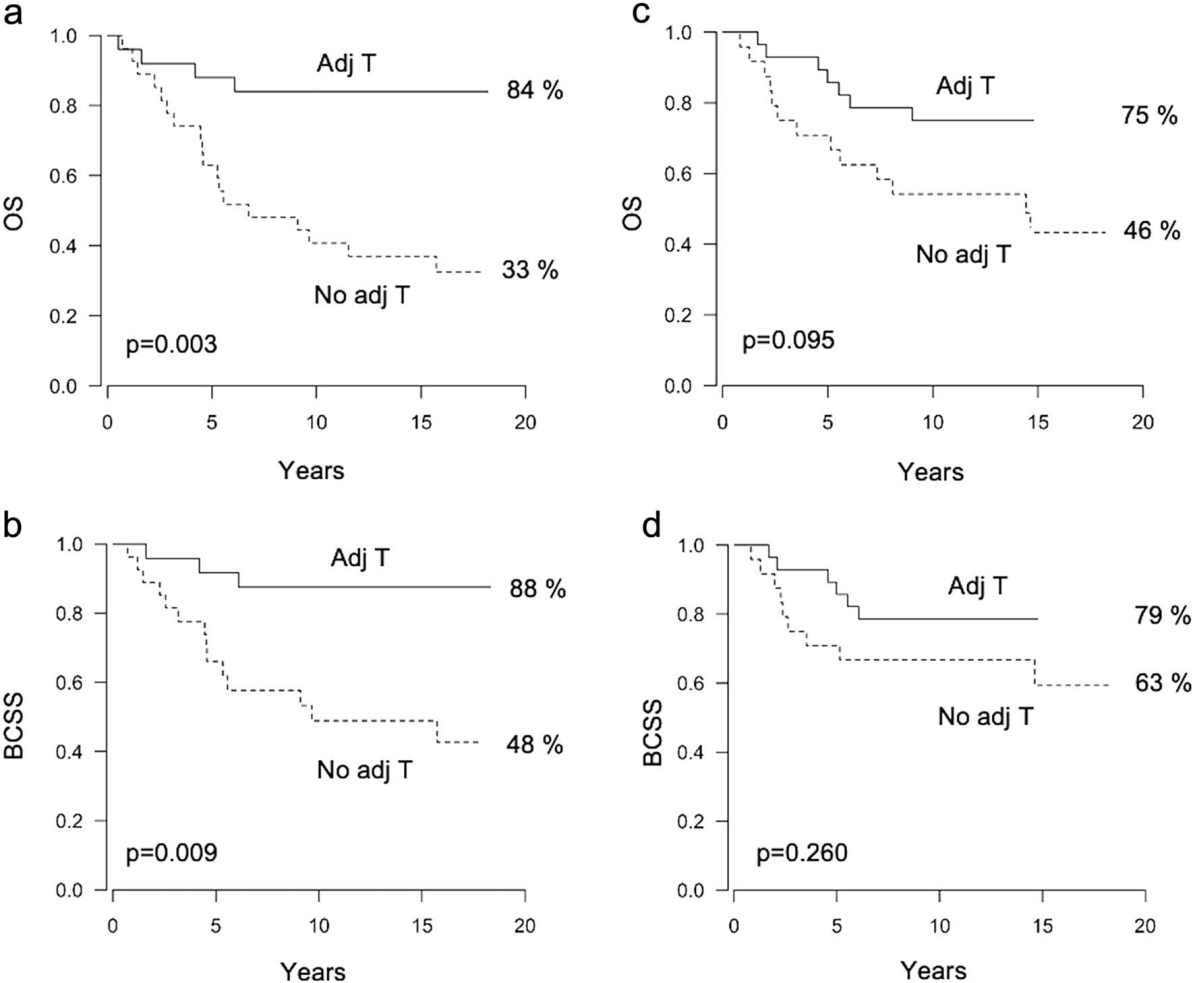
Survival rates according to the use of adjuvant trastuzumab (adj T) and CD8 + mTILs/CD68 + TAMs ratio. Kaplan–Meier curves showing overall survival (OS) and breast cancer-specific survival (BCSS) according to the administration of adjuvant trastuzumab in patients with a high CD8 + mTILs/CD68 + TAMs ratio (panels **a**, **b**), and those with a low CD8 + mTILs/CD68 + TAMs ratio (panels **c**, **d**)

The patients with a high CD8 + mTILs/CD68 + TAMs ratio (n = 52) benefitted considerably from adjuvant trastuzumab, as 84% of cases receiving trastuzumab but only 33% of the patients not treated with this drug were alive at the end of follow-up time (HR 0.19, 95% CI 0.06–0.56, p = 0.003) (Fig. 3a). Similarly, the corresponding values for BCSS were 88% vs. 48% (HR 0.19, 95% CI 0.054–0.66, p = 0.009) (Fig. 3b). Among the patients with a low CD8 + mTILs/CD68 + TAMs ratio (n = 52) the differences in OS and BCSS were not statistically significant; 75% with trastuzumab were alive compared to 46% not treated with trastuzumab (HR 0.45, 95% CI 0.18–1.15 p = 0.095) (Fig. 3c), and the corresponding values for BCSS were 79% vs. 63% (HR 0.55, 95% CI 0.19–1.57, p = 0.260) (Fig. 3d). However, the interaction between CD8 + mTILs/CD68 + TAMs ratio and trastuzumab therapy was not statistically significant. A high CD8 + mTILs/CD68 + TAMs ratio was associated with a higher tumor grade (p = 0.026) while there were no significant associations with other standard histopathological factors (data not shown). Examples of low and high CD8 + mTIL and CD68 + TAM stainings are shown in Supplementary Figure S1.

### Predictive value of CD8 + mTILs/FoxP3 + TILs ratio

**Fig. 4:**
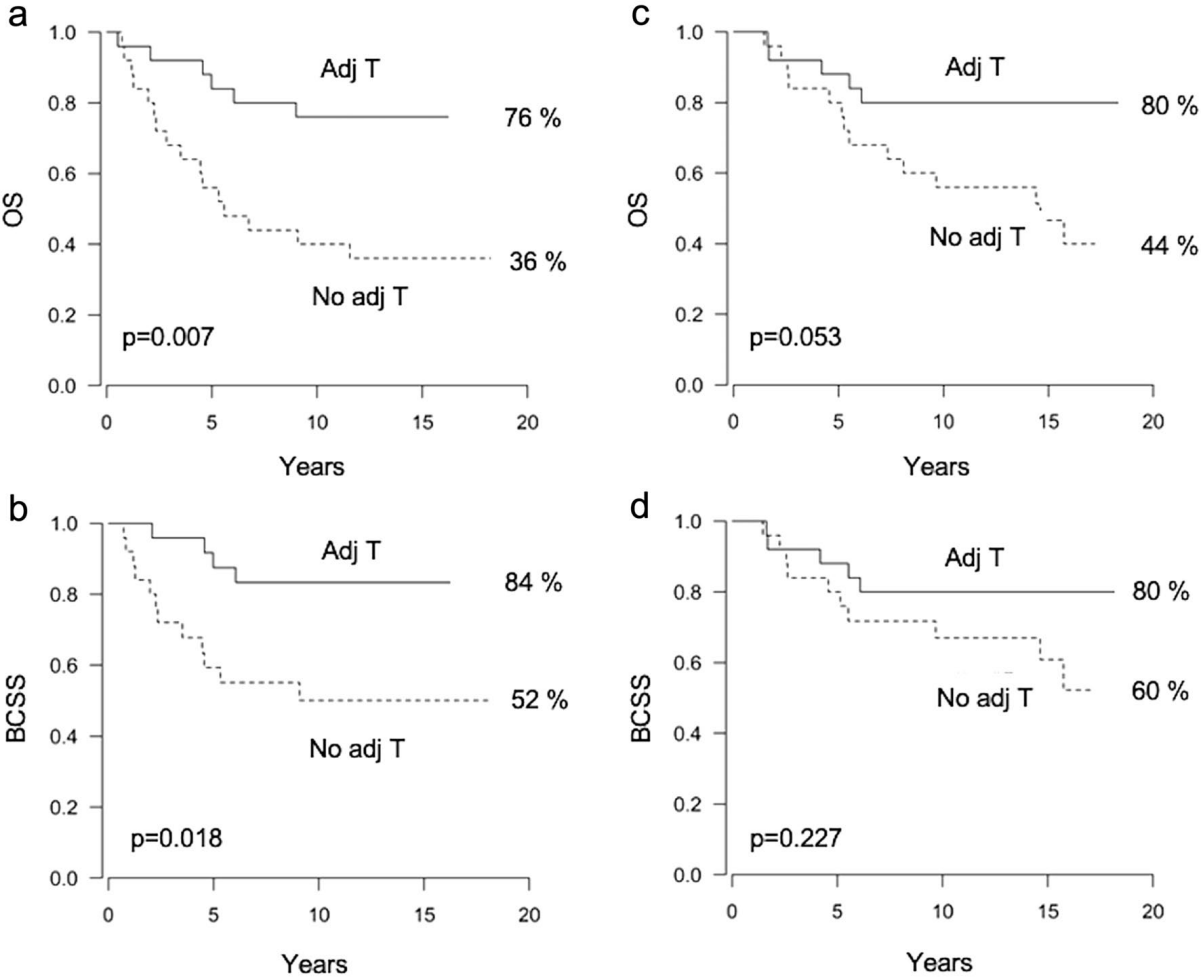
Survival rates according to the use of adjuvant trastuzumab (adj T) and CD8 + mTILs/FoxP3 + TILs ratio. Kaplan–Meier curves showing overall survival (OS) and breast cancer-specific survival (BCSS) according to the administration of adjuvant trastuzumab in patients with a high CD8 + mTILs/FoxP3 + TILs ratio (panels **a**, **b**), and those with a low CD8 + mTILs/FoxP3 + TILs ratio (panels **c**–**d**)

The patients with a high CD8 + mTILs/FoxP3 + TILs ratio (n = 50) gained a significant benefit from adjuvant trastuzumab therapy (76% vs. 36%, HR 0.27, 95% CI 0.11–0.70, p = 0.007 for OS and 84% vs. 52%, HR 0.25, 95% CI 0.08–0.79, p = 0.018 for BCSS) (Fig. 4a–b). For patients with a low CD8 + mTILs/FoxP3 + TILs ratio (n = 50), the differences in OS and BCSS did not reach statistical significance (80% vs. 44%, HR 0.36, 95% CI 0.13–1.02, p = 0.053 and 80% vs. 60%, HR 0.51, 95% CI 0.17–1.52, p = 0.227, respectively) (Fig. 4c–d). The interaction between CD8 + mTILs/FoxP3 + TILs ratio and trastuzumab therapy was not statistically significant. FoxP3 + TILs or CD8 + mTILs as single factors, or CD8 + mTILs/CD163 + TAMs ratio did not exhibit a predictive role in adjuvant trastuzumab therapy (data not shown).

### Cox multivariate survival analysis

In the COX multivariate analysis including the standard prognostic factors i.e., tumor size (T2-4 vs. T1), nodal status (N1-3 vs. N0), grade, ER/PR-status, and adjuvant trastuzumab therapy, the significant prognostic factors for OS were adjuvant trastuzumab (HR 0.29, 95% CI 0.15–0.55, p < 0.001) and tumor size (HR 2.06, 95% CI 1.12–3.78, p = 0.020).

## Discussion

In this cohort of 139 HER2 + breast cancer cases, we found that the numbers of FoxP3 + TILs and CD8 + mTILs correlated positively with each other and with TAMs. While the numbers of FoxP3 + TILs correlated positively with both CD68 + and CD163 + TAMs, the CD8 + mTILs count was associated only with CD68 + TAMs. In the HER2 + Luminal B subgroup, high numbers of FoxP3 + TILs were associated with a shorter DFS. Even though there was no statistically significant interaction between CD8 + mTILs/CD68 + TAMs ratio and trastuzumab therapy, the benefit from adjuvant trastuzumab seemed to be especially evident among the patients with a high CD8 + mTILs/CD68 + TAMs ratio as 84% of cases receiving trastuzumab but only 33% of the patients not treated with this drug were alive at the end of follow-up time. Among the patients with a low CD8 + mTILs/CD68 + TAMs ratio the differences in OS and BCSS according to the use of adjuvant trastuzumab were not statistically significant.

The positive correlation between the FoxP3 + TIL count and the CD68 + and CD163 + TAM counts may reflect the existence of a pro-tumoral microenvironment in HER2 + breast cancer, as Tregs and especially the M2-like (CD163 +) TAMs, contribute to the creation of inflammatory conditions promoting tumor progression [10, 13]. On the other hand, the positive correlation between FoxP3 + TILs and CD8 + mTILs may reflect some kind of balance between the immunoregulatory and immune activating cells, a finding in line with previous publications [20, 21]. Interestingly, the CD8 + mTIL count correlated positively only with the number of CD68 + TAMs, but not with CD163 + TAMs (M2-like), possibly due to the rather opposite roles of CD8 + TILs and M2-like TAMs in mediating the inflammatory responses. This finding is at odds with two other studies, where a high level of CD8 + TILs was found to positively associate with the M2-like TAM count [22, 23]. However, the breast cancer cases in those studies were mostly HER2-negative, which may explain the difference in the results, and support the hypothesis that the infiltration by immune cells and their immunological activity may vary according to the breast cancer subtype. A more comprehensive understanding of immunological factors in the complex tumor microenvironment would be advantageous for unravelling the mechanisms underpinning carcinogenesis in aggressive breast cancer subtypes such as HER2 + breast cancer.

Even though TILs in general have been linked with a better outcome in HER2 + breast cancer [7–9], the role of different TIL subtypes is less well defined [24–27]. In addition, the immunogenicity seems to vary between different HER2 + breast cancer subtypes e.g., ER-negative cases usually present greater infiltration of TILs as compared to ER-positive cases [28]. Here, we did not find any associations between survival and the evaluated immune cells in the total patient population. However, in HER2 + Luminal B breast cancer, a high FoxP3 + TIL count did associate with a shorter DFS, whereas there were no statistically significant associations in the HER2 + /HR- subgroup. Indeed, it has been suggested that the prognostic role of FoxP3 + and CD8 + TILs may be influenced by HR-status [10, 26, 29]. According to some investigators, ER-positive breast cancer seems to be the subgroup with a survival disadvantage from a high FoxP3 + TIL count and one study reported that among HER2 + /ER- patients, a high FoxP3 + TIL count might even improve the outcome [26, 30]. Estrogenic signaling is known to interact with immune activity and it has been suggested that immune activity might be reduced in HER2 + /HR + breast cancer [28]. The results of the present study together with these previous findings highlight the importance of recognizing that HER2 + Luminal B breast cancer and HER2 + /HR- breast cancer are two different entities. Considering the heterogeneous nature of HER2 + breast cancer and the complexity of the interplay between the tumor and the immune system, it is evident that more work is required to clarify the prognostic role of TILs in different HER2 + subtypes.

Our HER2 + breast cancer patient cohort is unique because half of the patients were treated before the year 2005, i.e., before adjuvant trastuzumab became included in the international guidelines. This allowed us to study the possible predictive value of the investigated immune cells in trastuzumab therapy. Neither FoxP3 + TILs nor CD8 + mTILs as single factors exhibited a predictive role on its own. As CD8 + TILs mediate rather opposite functions than TAMs and FoxP3 + TILs, we hypothesized that the ratios of CD8 + mTILs with TAMs and FoxP3 + TILs may be important for the efficacy of trastuzumab. Indeed, the patients with a high CD8 + mTILs/CD68 + TAMs ratio gained a major benefit from adjuvant trastuzumab whereas among the patients with a low CD8 + mTILs/CD68 + TAMs ratio the differences in OS and BCSS according to the use of adjuvant trastuzumab were not statistically significant. However, there was no statistically significant interaction between CD8 + mTILs/CD68 + TAMs ratio and trastuzumab therapy. We hypothesize that the patients benefit from adjuvant trastuzumab in both groups, but the survival advantage may be more evident if CD8 + mTILs/CD68 + TAMs ratio is high. In fact, the outcome of the patients treated without trastuzumab seemed to be even worse among the patients with a high CD8 + mTILs/CD68 + TAMs ratio than among those with low. In addition, the high CD8 + mTILs/CD68 + TAMs ratio was associated with a higher tumor grade. These results together suggest that a high CD8 + mTILs/CD68 + TAMs ratio is somehow associated with the aggressiveness of the disease, but the tumor tissue may simultaneously be especially sensitive to trastuzumab therapy. Interestingly, it has been previously shown in a mouse model that CD8 + TILs were essential for the efficacy of trastuzumab, but the removal of TAMs also seemed to be necessary [31, 32]. In human breast cancer, Loi et al. (2014) have reported that a high level of TILs predicted increased efficacy of trastuzumab therapy in early HER2 + breast cancer [4]. On the contrary, in another study conducted by Perez et al. (2016), high TILs appeared to predict lack of trastuzumab benefit [5]. Even though we found no statistically significant interaction between CD8 + mTILs/FoxP3 + TILs ratio and trastuzumab therapy, we hypothesize that in addition to the total TIL count also the numbers of the different lymphocyte subtypes are important. It was recently reported that a B-cell-related gene expression profile was superior to TILs in predicting the outcome of HER2 + breast cancer patients [33], highlighting the complexity of the regulation of immune responses. Furthermore, it should be noted that in our study, the individuals receiving adjuvant trastuzumab therapy also received chemotherapy more often, which may confound the interpretation of the results, as chemotherapy may also exert immunomodulatory effects.

The FoxP3 + TIL count, likewise the previously evaluated CD68 + and CD163 + TAMs, were analyzed by a microscope in the traditional manner. However, CD8 + TILs were assessed with a novel digital image analysis method, QuPath, which makes it possible to count a large number of cells in substantial areas of the tumor’s mass. The tumor margin was selected as an optimal area for evaluation, because CD8 + TILs are often predominantly localized at the tumor margin and it is the invasive border of carcinoma which may be crucial for the tumor’s spread. Previously, a study utilizing whole section slides for TILs, which were assessed with QuPath in cases of HER2-negative breast cancer, showed that the density of CD8 + TILs was higher in the invasive margin as compared to the tumor center, and the scoring results with QuPath correlated strongly with the manual counting values [34].

It is crucial to improve our understanding of the immune responses present in the tumor microenvironment if we are to develop novel therapies for HER2 + breast cancer. Immuno-oncological treatments, such as programmed death-1 (PD-1) and programmed death ligand-1 (PD-L1) inhibitors, are already in clinical trials for HER2 + breast cancer. In the present study, the correlations found between TILs and TAMs in the tumor microenvironment provide novel information of the immunological landscape in HER2 + breast cancer. In addition, a high FoxP3 + TIL count was associated with an inferior outcome in patients with HER2 + Luminal B breast cancer, but not in the HER2 + /HR-negative subgroup, highlighting the difference between these subgroups. Furthermore, the patients with a high CD8 + mTILs/CD68 + TAMs ratio gained a major benefit from adjuvant trastuzumab, suggesting that the inflammatory conditions in the tumor microenvironment may influence the efficacy of trastuzumab, but further studies addressing this question are needed. In the future, especially in the era of immuno-oncological treatments, it is important that we gain a better understanding of the complex immune landscape in the tumor microenvironment in order to improve the outcomes of HER2 + breast cancer patients.

## Supplementary Information

Below is the link to the electronic supplementary material.

Supplementary file1 (TIFF 16859 KB)—**Fig. S1** Examples of CD8+ mTIL and CD68+ TAM stainings. The figure shows examples of breast cancer sections with low CD8+ mTILs (panel a), high CD8+ mTILs (panel b), low CD68+ TAMs (panel c) and high CD68+ TAMs (panel d)

## Data Availability

The data are not publicly available due to privacy and ethical restrictions. The data that support the findings of this study may be available from the corresponding author on reasonable request and with required permissions.
