# Supplementary figures and images for: The prognostic and predictive role of tumor-infiltrating lymphocytes (FoxP3 + and CD8 +) and tumor-associated macrophages in early HER2 + breast cancer
Source: Breast Cancer Res Treat. 2023 Jul 10;201(2):183–92. doi: 10.1007/s10549-023-07017-8 (PMC10361875; doi:10.1007/s10549-023-07017-8)

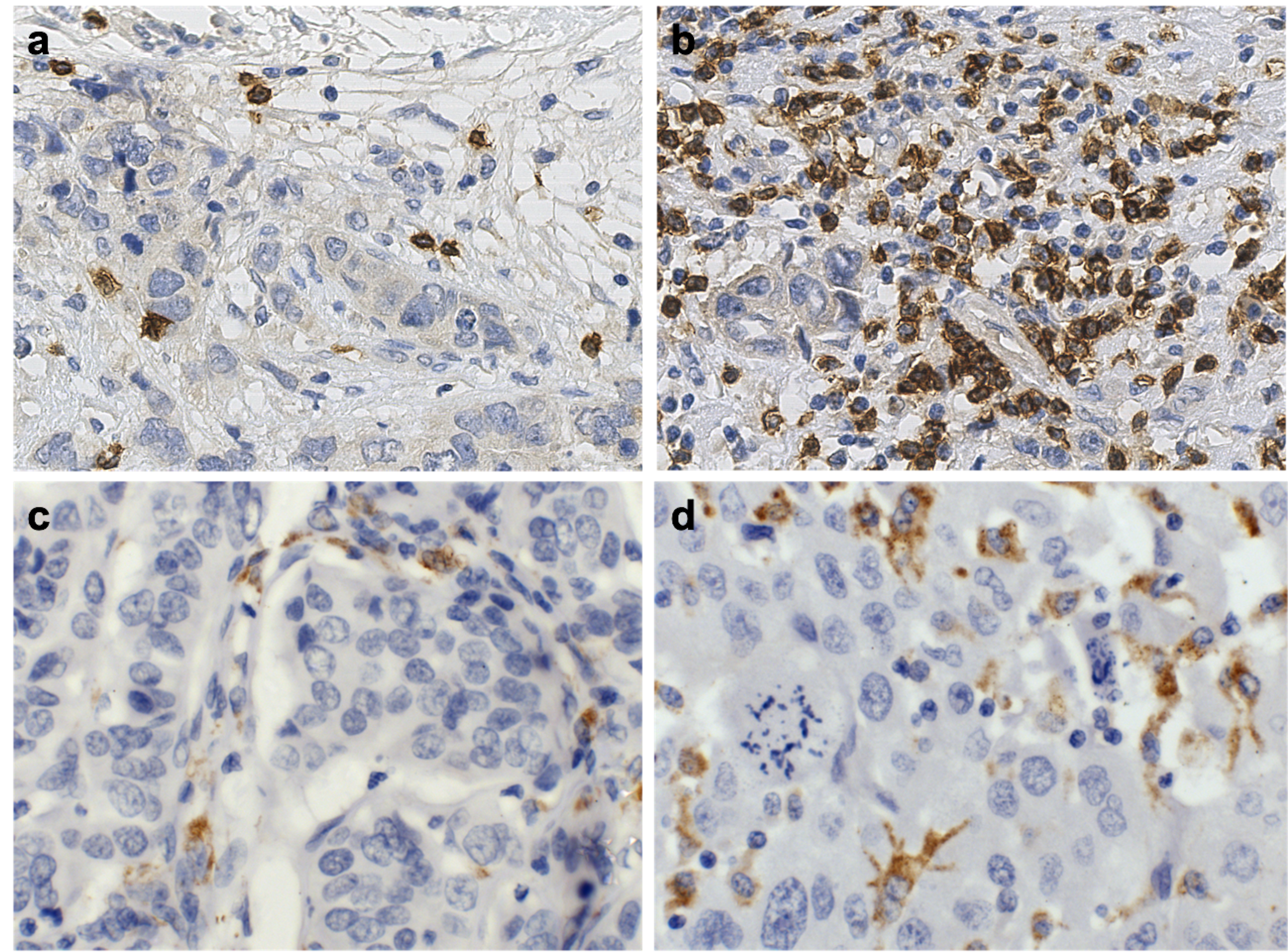

Supplement: Supplementary file 1 — Supplementary file1 (TIFF 16859 KB)—Fig. S1 Examples of CD8+ mTIL and CD68+ TAM stainings. The figure shows examples of breast cancer sections with low CD8+ mTILs (panel a), high CD8+ mTILs (panel b), low CD68+ TAMs (panel c) and high CD68+ TAMs (panel d) [file 10549_2023_7017_MOESM1_ESM.tiff]
